# Fluorescent *Tobacco mosaic virus*-Derived Bio-Nanoparticles for Intravital Two-Photon Imaging

**DOI:** 10.3389/fpls.2015.01244

**Published:** 2016-01-13

**Authors:** Annette Niehl, Florence Appaix, Sonia Boscá, Boudewijn van der Sanden, Jean-François Nicoud, Frédéric Bolze, Manfred Heinlein

**Affiliations:** ^1^Institut de Biologie Moléculaire des Plantes (IBMP-UPR2357), Centre National de la Recherche ScientifiqueStrasbourg, France; ^2^Two-Photon Microscopy Platform, Grenoble Institut des Neurosciences, Institut National de la Santé et de la Recherche Médicale U836, Université Grenoble AlpesGrenoble, France; ^3^Clinatec, Institut National de la Santé et de la Recherche Médicale UA 01Grenoble, France; ^4^Laboratoire de Conception et Application de Molécules Bioactives, UMR 7199 Centre National de la Recherche Scientifique-Université de StrasbourgIllkirch, France

**Keywords:** viral nanoparticles, *Tobacco mosaic virus*, two-photon microscopy, intravital imaging

## Abstract

Multi-photon intravital imaging has become a powerful tool to investigate the healthy and diseased brain vasculature in living animals. Although agents for multi-photon fluorescence microscopy of the microvasculature are available, issues related to stability, bioavailability, toxicity, cost or chemical adaptability remain to be solved. In particular, there is a need for highly fluorescent dyes linked to particles that do not cross the blood brain barrier (BBB) in brain diseases like tumor or stroke to estimate the functional blood supply. Plant virus particles possess a number of distinct advantages over other particles, the most important being the multi-valency of chemically addressable sites on the particle surface. This multi-valency, together with biological compatibility and inert nature, makes plant viruses ideal carriers for *in vivo* imaging agents. Here, we show that the well-known *Tobacco mosaic virus* is a suitable nanocarrier for two-photon dyes and for intravital imaging of the mouse brain vasculature.

## Introduction

Viruses are intensely studied since *Tobacco mosaic virus* (TMV) was found to be the causing agent of mosaic disease in tobacco (Harrison and Wilson, 1999; Scholthof, 2008). For the vast majority of the about 130 years after this finding, plant viruses have been investigated with the aim to understand and control virus-induced diseases in agricultural crops. As a result of these studies we have learned about the mechanisms of the disease processes and the fundamental nature of viruses, including the composition and structure of the viral particles, the viral replication cycle, and important virus:host interactions (e.g., Zaitlin and Palukaitis, 2000; Ding and Voinnet, 2007; Heinlein, 2015). Since about 30 years, plant viruses are also objects of biotechnological approaches, which led to a broad range of applications, from crop improvement to protein production systems. Plant virus systems have been particularly attractive for the production of recombinant proteins, including biopharmaceuticals, vaccines, and industrial proteins (Scholthof et al., 1996; Pogue et al., 2002; Cañizares et al., 2005; Grill et al., 2005; Gleba et al., 2007). The rod-shaped TMV and the icosahedral *Cowpea mosaic virus* (CPMV) are among the major vectors for plant-supported mass production of therapeutic peptides and proteins (Haynes et al., 1986; Gallie et al., 1987; Turpen, 1999; Smith et al., 2009; Sainsbury et al., 2010).

More recently, plant and other viruses became recognized as useful templates and building blocks for the development of nanotechnological applications (Steinmetz, 2010). The size of viral particles indeed falls into the nanometer range and thus they can be used for a variety of applications to create novel nano-sized materials with distinct properties. For example, the rod-shaped particles of TMV can be used as templates for mineralization and metallization reactions and the fabrication of highly ordered hybrid materials, even functional devices and ferrofluids (Dujardin et al., 2003; Flynn et al., 2003; Suci et al., 2006; Tseng et al., 2006; Wu et al., 2010; Atanasova et al., 2011; Chen et al., 2011; Lee et al., 2012). Moreover, since virus-derived nanoparticles represent naturally occurring nanomaterials that are both biocompatible and biodegradable, they are also being developed for biomedical applications. Here, plant viruses are particularly attractive since, unlike animal or human viruses, they do not cause diseases in humans. To establish the desired properties for biomedical use, viral nanoparticles (VNPs) can be designed and engineered using both genetic and chemical protocols. By both chemical and genetic manipulation, the viral coat can be tailored to a desired cell or tissue type, imaging modality, or therapeutic cargo. Its multivalent nature enables the incorporation of multiple functionalities, for example targeting ligands and imaging agents, on a single platform (Young et al., 2008), which may lead to potential applications in targeted imaging and therapy (Steinmetz, 2010).

The molecular structure and the biophysical properties of the rod-shaped TMV particle are well known (Alonso et al., 2013). The particle is about 300 nm in length and 18 nm in diameter, with a 4-nm wide inner channel. It has a mass of 39600 kDa and consists of 2130 viral coat protein (CP) subunits encapsidating a 6.7 kb long RNA in a helical arrangement. The CP consists of 158 amino acids and has a calculated mass of 17623.7 Da. Each CP subunit offers several accessible sites for chemical modification at the outer and inner surfaces and the possibility of insertion of peptides for surface display without necessarily affecting assembly or infectivity of the virus (e.g., Haynes et al., 1986; Fitchen et al., 1995; Smith et al., 2009). Virus particles can be purified in high yields from plants and are exceptionally stable allowing derivatization over a broad range of pH (3.5–9) and temperature (up to 90°C) in the presence of solvents such as ethanol or dimethyl-sulfoxide (Alonso et al., 2013). The virus particle readily assembles *in vitro* (Fraenkel-Conrat and Williams, 1955; Okada, 1986; Butler, 1999) with a short stretch of 432 nucleotides of its RNA (OAS, origin-of-assembly) being sufficient for assembly (Sleat et al., 1986). In the absence of RNA, the CP assembles into a 20 S nanoparticle or “disk,” which represents an assembly intermediate consisting of 34 CP subunits (Klug, 1999). Thus, CP monomers carrying different functional groups can be *in vitro* assembled into disks or particles of any desired length. The multivalent nature of TMV may offer the possibility for developing theranostic particles in which targeting and imaging capabilities are combined and which are designed for both the diagnosis and the treatment of diseases.

The capacity for the application of nanoparticles in the imaging and diagnosis of tissue alterations related to diseases depends on the use of fluorochromes and microscopes compatible with deep tissue imaging. This technology has been achieved with the development of two-photon (TP) laser scanning microscopy (TPLSM; Denk et al., 1990; Rubart, 2004) and of fluorophores optimized for two-photon absorption (TPA; Lincker et al., 2005; Pawlicki et al., 2009; Massin et al., 2013). TPLSM employs the excitation of fluorophores by photons in the infrared region for which biological material is transparent (Helmchen and Denk, 2005). Moreover, TPLSM produces background-free images with reduced photobleaching and photodamage since the simultaneous absorption of the two photons required for excitation is intrinsically restricted to the focal point of the excitation beam due to the non-linear nature of TPA (Rubart, 2004).

Here, we report the production of TMV particles carrying a two-photon fluorophore and their application as VNPs in intravital imaging of the mouse brain vasculature. The fluorescent signal emitted from the VNPs is stable and does not show any leakage into the surrounding tissues. The particles may have potential to contribute to the noninvasive detection and visualization of pathological alterations in the brain vasculature.

## Materials and methods

### BF3 synthesis

BF3-NCS has been prepared as described (Hayek et al., 2007a). BF3-NCS differs from compounds described in Hayek et al. (2007a) only by a shorter length of the oligoethylene glycol chains. The physico-chemical properties of BF3-NCS are: ^1^H-NMR, CDCl_3_; (δ ppm): 7.67–7.68 (dd, 2H, *J*_1_ = 0.88 Hz, *J*_2_ = 8.33 Hz), 7.50–7.46 (m, 4H), 7.11 (s, 2H), 7.06 (s, 2H), 6.80 (s, 2H), 6.78 (s, 2H), 4.24 (*t*, 4H, *J* = 4.8 Hz), 4.18 (*t*, 2H, *J* = 4.8 Hz), 4.03 (*t*, 2H, *J* = 6,3 Hz), 3.92 (s, 6H), 3.89 (*t*, 4H, *J* = 4.8 Hz), 3.87–3.64 (m, 22H), 3.57–3.54 (m, 6H), 3.38 (s, 3H), 3.37 (s, 6H), 2.04–1.85 (m, 8H), 0.70 (s, 10H). ^13^C-NMR, CDCl_3_; (δ ppm): 152.75; 152.37; 151.49; 140.58; 140.47; 138.32; 137.50; 136.68; 136.18; 136.10; 133.34; 133.06; 128.79; 128.68; 127.86; 127.74; 125.59; 120.62; 119.85; 106.19; 105.37; 103.44; 77.18; 72.37; 72.07; 71.92; 71.90; 71.89; 70.80; 70.74; 70.68; 70.66; 70.54; 70.51; 70.50; 70.45; 69.75; 68.83; 68.73; 58.99; 56.06; 55.93; 44.79; 26.99; 26.85; 17.15; 14.48. C_63_H_87_NO_15_S HRMS: m/z 1129.57952 (calc. m/z 1129.57963).

### Isolation of TMV particles

TMV particles were purified as described previously (Niehl et al., 2012). Briefly, leaves of TMV-infected *Nicotiana benthamiana* plants were ground in liquid N_2_ to fine powder. After addition of 10 mM sodium-phosphate buffer pH 7.2 containing 0.1% 2-mercaptoethanol, the viral particles were extracted with 1 volume of butanol/chloroform (1:1), precipitated with 4% polyethylene glycol (PEG) 8000, resuspended in 10 mM sodium-phosphate buffer pH 7.2 and cleared by centrifugation at 5000 × g for 10 min. After two cycles of precipitation in 4% PEG and clearance, the particles were resuspended in 10 mM sodium-phosphate buffer pH 7.2 and stored at −20°C until further use.

### Dye coupling and purification of TMV-BF3

300 μg of the purified viral particles per mg BF3-NCS dye were suspended in a sodium-phosphate buffer pH 7.2—DMSO mixture (3:1 V/V) and incubated in the dark at room temperature for 1.5 days. After coupling, the TMV-BF3 particles were separated from unbound dye and concentrated by centrifugation through Microcon YM-10 size exclusion columns (Millipore) using the manufacturer's instructions. The quality and quantity of the concentrated TMV-BF3 particles was assessed by transmission electron microscopy (TEM) following staining with 2% uranyl acetate and by SDS polyacrylamide electrophoresis (SDS-PAGE) using 15% SDS-polyacrylamide gels. Gels were imaged under UV light using an Ettan DIGE imager (GE Healthcare) equipped with a Sypro2 (excitation 390/20 nm; emission 595/25 nm) filter. After imaging, gels were stained with R250 Coomassie brilliant blue.

### Photometric measurements

All UV-visible absorption spectra were recorded on a Hitachi U3000 spectrophotometer in a dual beam mode using a matched pair of 1 × 1 cm quartz cells. Pure solvent was used as reference. Fluorescence emission spectra were measured with optically dilute solutions (Abs. < 0.15) in 1 × 1 cm cells using a Photon Technology International, Inc. (PTI) spectrofluorimeter.

### Two-photon absorption (TPA) measurements

The TPA cross-section spectra were obtained by up-converting fluorescence using a neodymium-doped yttrium aluminum garnet Nd:YAG-pumped optical parametric oscillator that produces 2.6 ns [full width at half maximum (FWHM)] pulses for excitation in the 450–650 nm spectral range and a Ti:sapphire femtosecond laser for excitation in the 700–900 nm range. This set-up does not allow TPA measurements between 650 and 700 nm. The excitation beam was collimated over the spectrophotometric cell length (10 mm). The fluorescence, was collected at 90° of the excitation beam and focused into an optical fiber connected to a spectrometer. The incident beam intensity was adjusted to ensure an intensity-squared dependence of the fluorescence over the whole spectral range. Calibration of the spectra was performed with *p*-bis(*o*-methylstyryl)benzene, for which the TPA cross section σ_2_ is 70 GM (Göppert-Mayer) at 570 nm (1 GM = 10^−50^ cm^4^ s / photon), and with Rhodamine B, for which the TPA spectrum at 700–900 nm is known (Xu and Webb, 1996).

### Two-photon fluorescence correlation spectroscopy (TP-FCS)

TP-FCS was performed with a home-built setup. TP excitation was provided by a Tsunami Ti:sapphire laser pumped with a Millennia V solid-state laser (Spectra-Physics, Mountain View, CA). 80 Mhz pulses of 100 fs were applied with a wavelength of 760 nm. Following passage through a beam expander, the infrared light was focused into the sample by a water-immersion Olympus objective (60 ×, NA = 1.2) mounted on an Olympus IX70 inverted microscope. The back aperture of the objective was slightly overfilled, creating a diffraction-limited focal spot. The sample and the reference dye were placed in eight wells of a Lab-Tek chambered cover glass (Nalge Nunc International, Rochester, NY) positioned in the X and Y directions by a motorized stage (Märzhäuser, Germany). The fluorescence from the samples was collected through the same objective and directed by a COWL750 dichroic mirror (Coherent, Orsay, France) toward a 50 μm diameter optical fiber coupled to an avalanche photodiode (SPCM 200 FC, EG&G, Canada). Residual infrared light was rejected by a BG39 Filter (Coherent). For FCS measurements, the normalized autocorrelation function (ACF), G(τ), of the fluorescence intensity fluctuations was calculated online by an ALV5000E digital correlator card (ALV, Langen, Germany). Calibration of the system was performed with a 50 nM tetramethylrhodamine (TMR) solution. Assuming a diffusion constant of 2.8 × 10^−10^ m^2^ s^−1^ (Clamme et al., 2003), the equatorial (r0) and axial (z0) radii of the focal volume were, respectively, 0.29 and 1.3 μm, giving an effective volume of 0.2 fL. Assuming a three-dimensional Gaussian distributed excitation intensity, the ACF curve for our sample was fitted as described by Clamme et al. (2003). The fitting curve for our sample corresponded to a bi-exponential function (y = Ae^ax+b^ + Be^cx+d^), thus reflecting the presence of two different species with very distinct molecular weights (BF3-coupled virus particles and free BF3 dye) in the solution.

### *In vivo* two-photon laser scanning microscopy (TPLSM)

In accordance with the policy of Grenoble Institute of Neuroscience (GIN) and the French legislation, experiments were done in compliance with the European Community Council Directive of November 24, 1986 (86/609/EEC). The research involving animals was authorized by the Direction Départementale des Services Vétérinaires de l'Isère—Ministère de l'Agriculture et de la Pêche, France and the Direction Départementale de la protection des populations—Préfecture de l'Isère-France (F. Appaix, PhD, permit number 38 09 39). All efforts were made to minimize the number of mice used and their suffering during the experimental procedure. CD1 Mice were housed in cages with food and water *ad libitum* in a 12 h light/dark cycle at 22 ± 1°C.

For *in vivo* TPLSM, the 4 months old CD1 mouse in the experiment was anesthetized using isoflurane (5% for induction and 1–2% during experiments) in a 70% air, 30% O_2_ gas mixture. Its body temperature was monitored with a rectal probe and maintained at 36°C using a heating blanket. The MouseOx system (STARR Life Sciences Corp.) was used for monitoring arterial O_2_ saturation, as well as the heart beat and breathing rate. A catheter (NeoflonTM, BD, USA) was inserted in the tail vein for intravenous (iv) injection of 100 μL of TMV-BF3 (50 mg/mL) in saline just before the imaging experiments. Sulphorhodamine B was diluted to 10 mg/mL in saline and 0.1 mL was injected 1 h after the iv injection of TMV-BF3 in order to control if the fluorescent TMV particles addressed the same brain microvessels as observed by small conventional dyes like Sulforhodamine B.

For intravital two-photon imaging of the cerebral vasculature, a craniotomy of 2–3 mm in diameter was performed with a surgical drill above the motor cortex and filled with ultrasound gel (the head was fixed in a homebuilt stereotactic frame). TPLSM was performed using a LSM 7 MP (Zeiss, Germany) equipped with a 20x water-immersion objective (NA 1.0; Zeiss) and ZEN 2010 software. The blue fluorescence light emission of the TMV-BF3 particles and the red fluorescence of the sulforhodamine B dye were simultaneously collected in the epifluorescence configuration using two photomultiplier tubes with a FF01 492/SP25 nm filter (Semrock, US) in front of the “blue PMT” and a FF01 617/73 nm filter (Semrock, US) in front of the “red PMT.” Laser excitation was performed at 800 nm using a Ti:Sapphire laser (Chameleon Vision II; Coherent, UK). All the TPLSM images were obtained with less than 50 mW laser power at the cortical surface. Most 3D TPLSM images were acquired as z-stacks with 607 × 607 μm size x-y direction and 2 μm step sizes between each z-focus plane. The 3D projections were performed with Fiji software (http://fiji.sc/Fiji) using the in-built “Z-Project” feature (http://fiji.sc/Z-functions) and the standard deviation projection method.

## Results

### Preparation of BF3-coupled TMV particles

**Figure 1:**
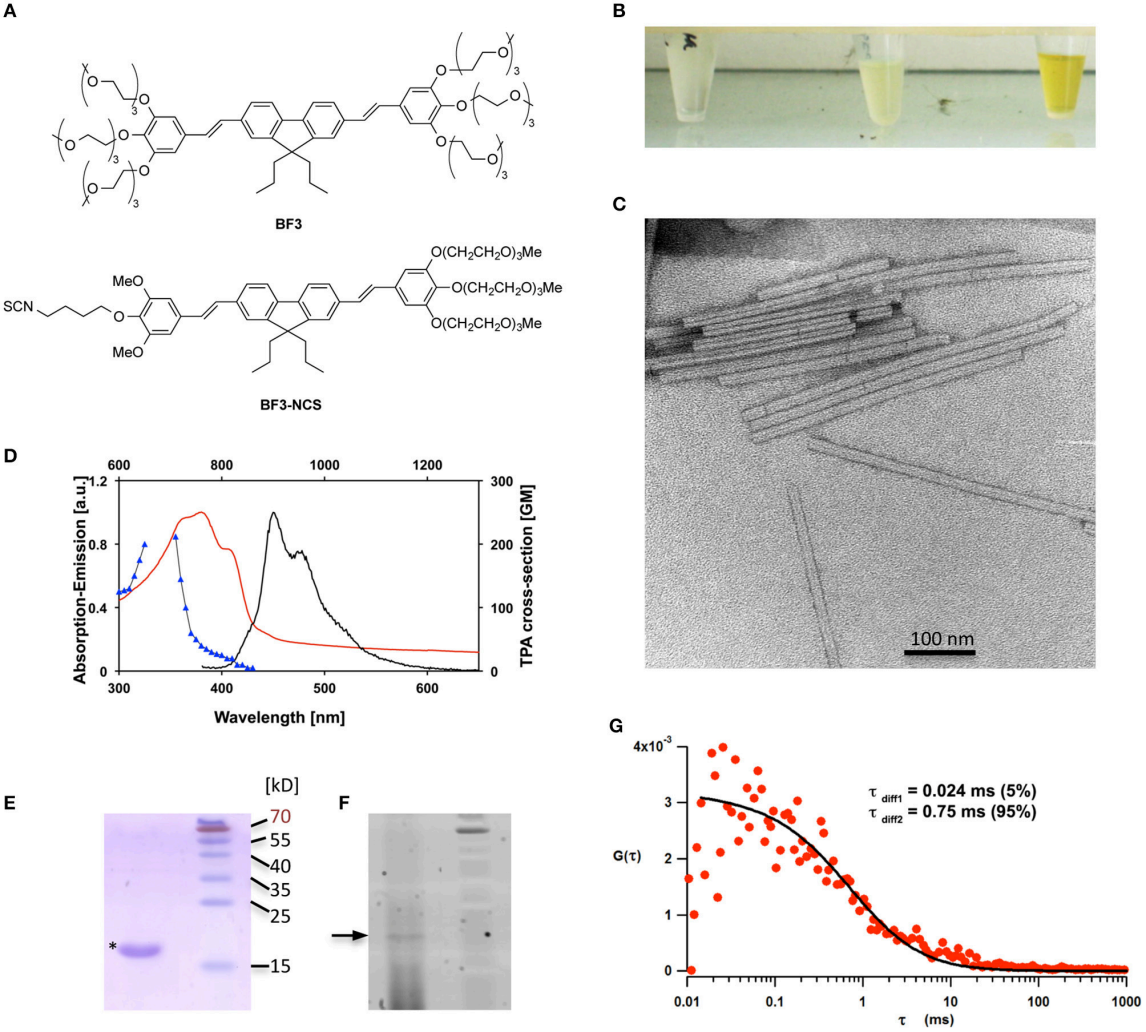
**Production and analysis of TMV-BF3 particles**. **(A)** Molecular formulae of BF3 and BF3-NCS. **(B)** Solutions containing purified TMV-particles before coupling (left), non-purified TMV particles after coupling (middle), and TMV particles size-purified after coupling (right). **(C)** EM image showing integrity of TMV particles after coupling. **(D)** Absorption (red) and emission (black) spectra of TMV-BF3 particles in phosphate buffer; two-photon excitation spectrum (blue) of BF3-NCS in water; a.u., arbitrary units; GM, Goeppert-Mayer units. **(E,F)** Electrophoretic analysis of the size-purified TMV-BF3 particles under denaturing conditions. **(E)** Coomassie blue-stained SDS-PAGE gel showing the presence of CP (17.6 kD, asterisk). **(F)** Same gel as in E but analyzed with a fluorescence scanner equipped with a sypro2 filter. A fluorescent band at approximately 18.5 kD is detected and confirms the presence of BF3-coupled CP. The orange dye conjugated to the 70 kDa protein of the size ladder (Thermo Scientific) also exhibits fluorescence under the illumination conditions used. The occurrence of fluorescence below the BF3-CP band suggests that the bond between BF3 and CP is partially sensitive to the denaturating SDS-PAGE conditions. **(G)** TP-FCS analysis of TMV-BF3 particles under native conditions showing a long diffusion time of 0.75 ms corresponding to the labeled viral particle (>95%) and a shorter time of 0.024 ms corresponding to free dye (<5%).

TMV particles isolated from infected *Nicotiana benthamiana* plants were coupled to BF3-NCS (Hayek et al., 2007a), a derivative of the two-photon-excitable fluorophore BF3 (Hayek et al., 2006; Figure 1A), a non-toxic molecule emitting blue fluorescence with an improved fluorescence quantum yield of 73% in water. BF3-NCS carries long oligoethyleneglycol side chains to provide increased water solubility and an *iso*-thiocyanate group (NCS) for chemical coupling (e.g., to −NH_2_ and −OH groups, i.e., the NH_2_ group at the N-terminus or the −OH group of tyr 139 of CP). As a one-dimensional (1D) conjugated molecule of the general symmetrical D–conjugated π system–D (D = electron donor group) structure, it shows a high TPA cross-section (Figure 1D).

Before conjugation, virus particles were purified from TMV-infected plants. The purity of the viral particle preparation was assessed by SDS-PAGE. The particles were then conjugated to BF3-NCS in a water/DMSO mixture for 36 h and purified by size exclusion centrifugation. As compared to the colorless solution that contains viral particles before coupling or the pale-yellow color of the solution containing coupled but non-purified particles, the solution containing the concentrated BF3-coupled viral particles was characterized by a strong yellow color (Figure 1B) and showed intense blue fluorescence in UV light. Following coupling and purification, the viral particle preparation maintained infectivity as was verified by the observation of local cell death lesions forming on leaves of hypersensitive *Nicotiana tabacum* NN tester plants (not shown). The typical rod-like shape and size dimension of the TMV particles was not affected by BF3-coupling as was confirmed by electron microscopy (EM; Figure 1C). 1-photon absorption and fluorescence emission spectra of the BF3-conjugated particles showed maxima at 382 and 452 nm, respectively. Moreover, the absorption was in agreement with the two-photon absorption peak of the free dye (Figure 1D). These values are similar to the spectra previously reported for the parent BF3 molecule (Hayek et al., 2007b). Analysis of the purified particles by SDS-PAGE revealed a fluorescent protein band running at higher molecular weight (approximately 18.5 kD) than the normal CP (17.6 kD), thus confirming successful conjugation (Figures 1E,F). The two-photon sensitivity as well as the purity of fluorescent particles were verified by two-photon fluorescence correlation spectroscopy (TP-FCS), which showed a long diffusion time of 0.75 ms corresponding to the labeled viral particle (>95%) and a shorter time of 0.024 ms, which corresponds to free dye (<5%) (Figure 1G).

### Application of TMV-BF3 particles for intravital imaging

**Figure 2:**
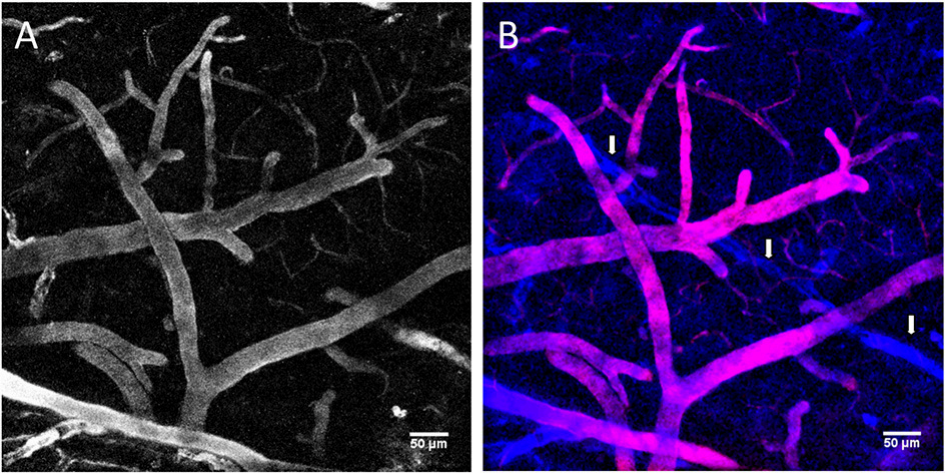
**Intravital imaging of the mouse brain vasculature with TMV-BF3 particles**. **(A)** Mouse brain vessels labeled with TMV-BF3 at 1 h after intravenous injection into the tail vein. **(B)** Same observation window as shown in **(A)** but after a second injection, this time with sulforhodamine B; blue, fluorescence emitted from TMV-BF3; red, fluorescence emitted from sulforhodamine B. The 3D projections were performed with Fiji software using the standard deviation projection method.

To determine the applicability of the dye-conjugated particles for *in vivo* TPLSM imaging, we injected 100 μL of the particle solution [50 mg/mL in saline (0.9% NaCl)] into the tail vein of an anesthetized 4 months-old CD1 mouse placed on the microscope stage and prepared by craniotomy. We then observed the cerebral vasculature at the surface of the motor cortex by TPLSM using a high numerical aperture, 20x water immersion objective and an excitation wavelength at 800 nm. The cerebral vasculature was readily visible by strong fluorescence with no obvious evidence for leakage until 1.5 h after iv injection (see Figures 2A,B). In previous studies (Hayek et al., 2007b), the free fluorescent dye diffused freely across the BBB in an 8 months old mouse within 20 min after injection. Acquisitions of z-stacks were used to reconstruct 3D animations that provide detailed views of the brain vasculature (Movie 1). To verify the pattern of blood vessels seen with TMV-BF3 by a different staining, the same mouse was post-injected (1 h after injection of TMV-BF3) into the tail vein with 100 μl of a sulforhodamine B solution (10 mg/mL). As shown in the overlay Figure 2B, at 1.5 h post-injection the pattern of vessels labeled with the red fluorescent dye (red) overlapped with the pattern of vessels labeled with TMV-BF3 (blue). However, there were also important exceptions of vessels that were labeled with TMV-BF3 but not or, only weakly, with sulforhodamine B (e.g., the vessel highlighted by arrows). This observation indicates that the TMV-BF3 particles partly blocked the blood perfusion in small brain vessels.

## Discussion

Our observations demonstrate that TMV particles labeled with a multi-photon absorbing fluorochrome can be used as a blood pool dye for deep tissue vascular imaging. Current agents for vascular imaging are based on liposomes, quantum dots, dextrans, lectins, antibodies, iron oxide particles, and nanospheres. Although each particle type has certain advantages, diverse problems in relation to stability, bioavailability, toxicity, chemical flexibility, and cost exist (Lewinski et al., 2008; Cheng et al., 2012; Adjei et al., 2014). Moreover, inorganic synthetic particles tend to aggregate and may cause toxic effects under physiological conditions (Kirchner et al., 2005). TMV particles represent natural materials that are both biocompatible and biodegradable and thus provide a safe platform for biomedical applications. Because of their anisotropic shape and their size, the particles may provide a sensitive probe to measure blood flow. In addition, large dyes also play a role as probes in other cases of brain diagnostics such as the optical mapping of damages to the BBB. The BBB consists of a tightly packed layer of endothelial cells that acts as a physical barrier between the blood vessels and the central nervous system and prevents many substances from diffusing across it (Rubin and Staddon, 1999). Disruption of the BBB causing leakage of substances from the blood vessels into the brain is a serious condition that occurs in many pathological conditions such as brain tumors (Jain, 1987; Dvorak et al., 1988; Hashizume et al., 2000), brain injuries (McDannold et al., 2008; Moretti et al., 2015), infections of the central nervous system (Lossinsky and Shivers, 2004), neurological diseases such as multiple sclerosis (Patel and Frey, 2015), Parkinson's diseases (Lee and Pienaar, 2014), or stroke (Gartshore et al., 1997; Latour et al., 2008). BBB openings allowing leakage can also be induced by radiotherapy or drugs (e.g., nicotine; Manda et al., 2010), or by physical factors such highly focused ultrasound (Yang et al., 2014) or blast-associated pressure waves (Kabu et al., 2015). The proper mapping of blood supply to brain regions affected by BBB leakage requires large dyes to label the leaky vessels without important diffusion into the extravascular space, which would otherwise lead to an overestimation of the functional blood volume supply and blur two-photon microscopic images (Maurin et al., 2011). Small dyes such as sulforhodamine B, but also the well-established blood pool dye 70 kDa rhodamine-labeled dextran with a hydrodynamic radius of 6 nm, and even the largest dextrans (200 kDa) with a hydrodynamic radius of 27 nm are known to diffuse across leaky vessels (Jain, 1987; Dvorak et al., 1988; Vérant et al., 2008; Maurin et al., 2011). Large dyes such as TMV-BF3 also have potential to contribute to the imaging of primary brain tumors, in particular glioblastomas, which are among the most therapy-resistant tumors. The combined application of both large dyes, such as TMV-BF3, and small diffusible dyes as probes for multi-photon deep tissue imaging of the healthy and diseased brain may represent a promising approach for exploring changes in transport barriers and for distinguishing between normal and functional vessels with a leaky BBB that are important for drug supply during tumor growth. VNPs such as TMV-BF3 may also be further developed to deliberately cross the BBB and thus to deliver therapeutics to the diseased brain (Meyers et al., 2013).

The application of TMV particles for vascular and other *in vivo* imaging applications will have to await further improvements. Our preliminary *in vivo* data indicate that large TMV particles may obstruct the blood perfusion in some micro-vessels probably due to the formation of aggregates at low blood flow. Thus, TMV particles of different sizes should be tested to optimize the particle size for specific *in vivo* biomedical applications. This is straightforward since CP assembles *in vitro* into small 34-mers (disks, 20 S particles) or, in the presence of RNA carrying the OAS, to larger particles with sizes determined by the size of RNA template (Fraenkel-Conrat and Williams, 1955; Okada, 1986; Butler, 1999; Klug, 1999). It should be considered also that TMV is ubiquitously present in the environment and that most if not all mammals and humans carry TMV-specific antibodies in their blood streams. Thus, although compatible with blood, TMV is usually cleared rapidly from blood by liver and spleen (Bruckman et al., 2014). However, this may actually provide an advantage over inorganic particles, as TMV particles prone to rapid clearance after application may prevent potential side effects. Recurrent administrations may prolong their application if needed. To increase the half-life of circulation of TMV within the blood vessels, the particle may also be coated with polyethylene glycol (PEG), which will minimize molecular interactions with the particle surface and thus recognition by the immune system (Raja et al., 2003; Steinmetz and Manchester, 2009; Bruckman et al., 2014). On the other hand, the coupling of BF3 to TMV may already increase TMV half-life in the blood, since it was shown that the grafting of a molecule similar in size to BF3 inhibited the elicitation of an antibody response (Wei et al., 2014). This effect could be enhanced by the presence of oligoethylene chains at the periphery of the fluorophore, thus rendering the particle even more furtive. It will have to be seen whether and in as much the circulation half-life, biodistribution, and clearance mechanisms of TMV-BF3 particles will be influenced by their final shape, size, and surface properties, as shown for other nanoparticles (Geng et al., 2007; Gratton et al., 2008; Li and Huang, 2008; Arnida et al., 2011; Longmire et al., 2011; Sa et al., 2012).

The utilization of plant VNPs for intravital vascular imaging was previously demonstrated with particles of *Cowpea mosaic virus* (CPMV; Lewis et al., 2006). However, unlike in our current study, one-photon dyes were used and fixation and sectioning were required for deep tissue imaging in adult mice. Moreover, the icosahedral particles of CPMV do not provide flexibility in particle design like TMV. Interestingly, the fluorochrome-tagged CPMV particles did not remain within vessels but entered the vessel endothelium. Although such feature may contribute to the long-term stability of the fluorescence signal, it also limits application in blood flow measurements and may also enhance the risk of leakage across the BBB. In addition to CPMV and TMV, many other plant viruses are being investigated as VNPs for applications in medicine (Steinmetz et al., 2010; Yildiz et al., 2010; Wen et al., 2012; Shukla et al., 2014). For example, CPMV and other plant viruses, like *Cowpea chlorotic mottle virus* (CCMV), and also bacteriophages such as MS2 and Qβ, are explored as platforms for the binding of several hundred contrast agents in order to improve the efficiency of magnetic resonance imaging (MRI; Liepold et al., 2007). Combining these or fluorescent plant VNPs (e.g., TMV-BF3) with specific targeting ligands and therapeutic agents such as drugs or peptides may lead to the development of novel cost-effective tools for *in vivo* imaging and treatment approaches that may eventually revolutionize the current concepts of diagnosis and therapy.

## Author contributions

AN, FB, JFN, BS, and MH conceived and designed the work; AN, SB, FA, and FB performed the acquisition and analysis of the data; AN, FB, JFN, BS, and MH interpreted the data; AN, FA, SB, BS, JFN, FB, and MH drafted the work; FB, BS, and MH wrote the manuscript and revised it critically for important intellectual content; AN, FA, SB, BS, JFN, FB, and MH approved the final version to be published and agree to be accountable for all aspects of the work.

## Funding

This work was performed with financial support by the University of Strasbourg Institute of Advanced Study (USIAS) to MH. The intravital microscopy platform at the Institute of Neuroscience Grenoble received funding of the French national infrastructure GIS-IBiSA.

### Conflict of interest statement

The authors declare that the research was conducted in the absence of any commercial or financial relationships that could be construed as a potential conflict of interest.

## Supplementary material

Movie 1 **3D-animated part of the TMV-BF3 labeled mouse brain vasculature**.
